# Prospective Studies on the Risk of Rheumatoid Arthritis: The European Risk RA Registry

**DOI:** 10.3389/fmed.2022.824501

**Published:** 2022-02-22

**Authors:** Paul Studenic, Aase Hensvold, Arnd Kleyer, Annette van der Helm-van Mil, Arthur G. Pratt, Daniela Sieghart, Gerhard Krönke, Ruth Williams, Savia de Souza, Susanne Karlfeldt, Martina Johannesson, Niels Steen Krogh, Lars Klareskog, Anca I. Catrina

**Affiliations:** ^1^Department of Medicine Solna, Division of Rheumatology, Karolinska Institutet, Stockholm, Sweden; ^2^Department of Internal Medicine 3, Division of Rheumatology, Medical University of Vienna, Vienna, Austria; ^3^Academic Specialist Centre—Stockholm Health Care Services, Centre for Rheumatology, Stockholm, Sweden; ^4^Universitätsklinikum Erlangen, Deutsches Zentrum Immuntherapie, Friedrich-Alexander University Erlangen-Nuremberg, Erlangen, Germany; ^5^Department of Internal Medicine 3 - Rheumatology and Immunology, Universitätsklinikum Erlangen, Friedrich-Alexander University Erlangen-Nuremberg, Erlangen, Germany; ^6^Department of Rheumatology, Leids Universitair Medisch Centrum, Leiden, Netherlands; ^7^Department of Rheumatology, Erasmus Medical Centre, Rotterdam, Netherlands; ^8^Translational and Clinical Research Institute, Newcastle University, Newcastle upon Tyne, United Kingdom; ^9^Musculoskeletal Services Directorate, The Newcastle upon Tyne Hospitals NHS Foundation Trust, Newcastle upon Tyne, United Kingdom; ^10^Centre for Rheumatic Diseases, King's College London, London, United Kingdom; ^11^ZiteLab, Frederiksberg, Denmark; ^12^Rheumatology Section, Theme Inflammation and Infection, Karolinska University Hospital, Stockholm, Sweden

**Keywords:** rheumatoid arthritis, prevention, database, multi-center study, observational

## Abstract

**Background:**

The accumulation of risk for the development of rheumatoid arthritis (RA) is regarded as a continuum that may start with interacting environmental and genetic factors, proceed with the initiation of autoimmunity, and result in the formation of autoantibodies such as anti-citrullinated peptide antibodies (ACPA). In parallel, at-risk individuals may be asymptomatic or experience joint pain (arthralgia) that is itself non-specific or clinically suspicious for evolving RA, even in the absence of overt arthritis. Optimal strategies for the management of people at-risk of RA, both for symptom control and to delay or prevent progression to classifiable disease, remain poorly understood.

**Methods:**

To help address this, groups of stakeholders from academia, clinical rheumatology, industry and patient research partners have collaborated to advance understanding, define and study different phases of the at-risk state. In this current report we describe different European initiatives in the field and the successful effort to build a European Registry of at-risk people to facilitate observational and interventional research.

**Results:**

We outline similarities and differences between cohorts of at-risk individuals at institutions spanning several countries, and how to best combine them within the new database. Over the past 2 years, besides building the technical infrastructure, we have agreed on a core set of variables that all partners should strive to collect for harmonization purposes.

**Conclusion:**

We emphasize to address this process from different angles and touch on the biologic, epidemiologic, analytic, and regulatory aspects of collaborative studies within a meta-database of people at-risk of RA.

## Introduction

**Figure 1:**
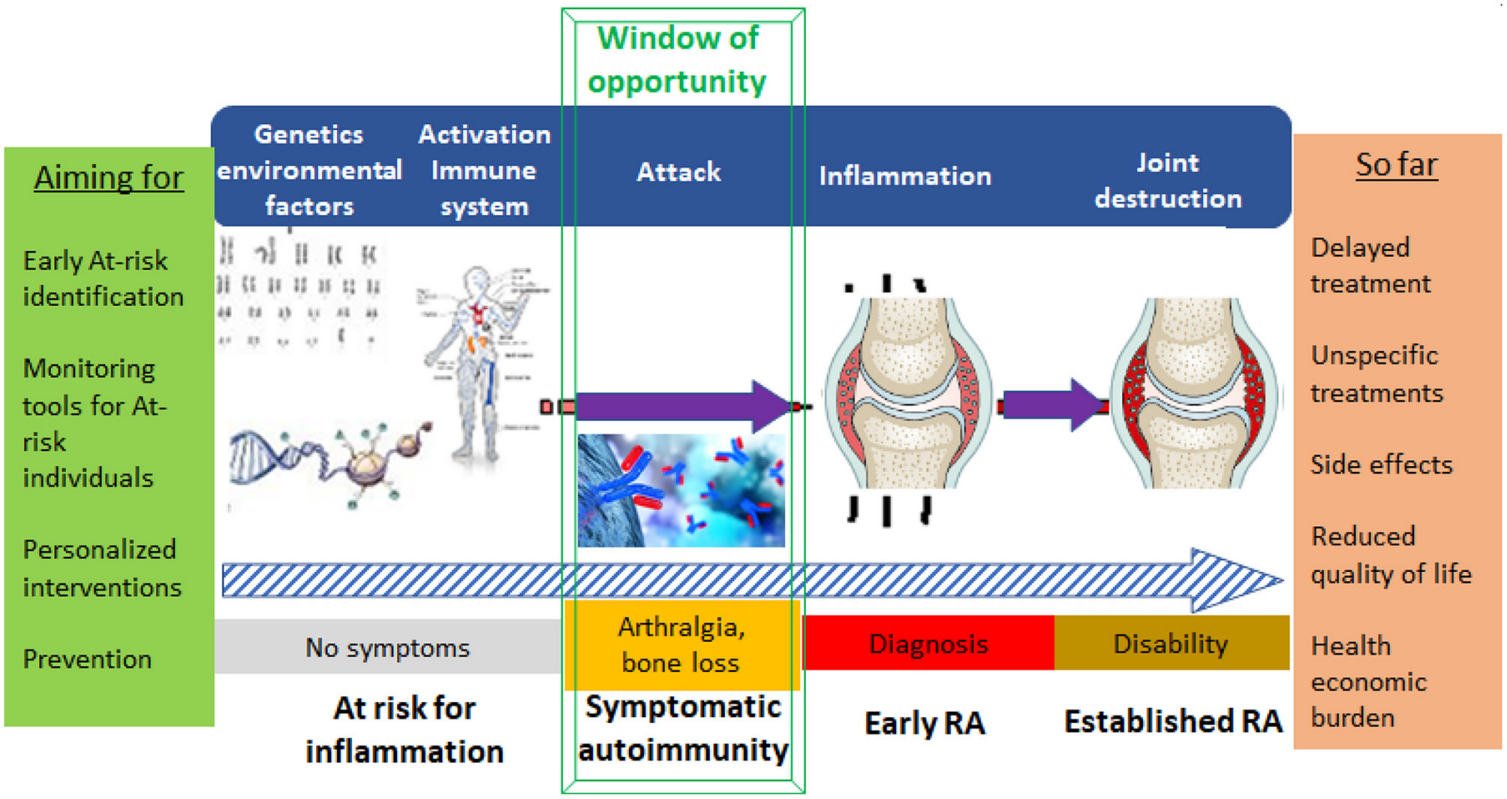
Visualizing the development of RA and key aims in future healthcare management of people in different stages/phenotypes at-risk of developing arthritis/RA.

The current classification criteria for rheumatoid arthritis (1, 2) published in 2010 make it possible to better define patients at an earlier stage of their disease course in comparison to 1987 ACR criteria. Understanding of how the natural history of RA can be temporally subdivided is no longer limited to early and established RA, but also acknowledges our growing understanding of the pre-disease phases (3–5). Further insights into the development of RA have been formalized into an agreed concept that constitutes the basis for the definition of the criteria of clinically suspect arthralgia (CSA) amongst individuals at risk to develop RA (6). In some individuals certain environmental and/or genetic risk factors can lead to the awakening of systemic autoimmunity, the formation of autoantibodies and musculoskeletal (MSK) symptoms, before the onset of arthritis (7–11). In the next step, patients that eventually develop clinically apparent arthritis might then be classified at some point as having RA. Based on this concept (Figure 1), it is of importance for further understanding of the gradual emergence of RA and for the development of preventive strategies for arthritis that cohorts of individuals with musculoskeletal symptoms and RA-associated immunity are created. When not considering the ACR/EULAR classification criteria for RA (12), the decision when an individual “at-risk” has formally become a patient with arthritis can be shifted along this above explained continuum. The identification or naming of arthritis is also depending on assessment measures used in the work-up of these individuals at-risk, since arthritis might be identified using additional (imaging) techniques at early points (13).

The Rheuma Tolerance for Cure (RTCure) consortium started in September 2017 aiming to change the treatment paradigm from late and non-specific to early and specific (Figure 1) and to improve understanding of the at-risk phases of RA and factors determining the transition toward disease. One major task for the consortium is to develop and populate a longitudinal register with defined inclusion criteria, methods for surveillance of included individuals and defined outcomes (arthritis). This collaborative and harmonized “pool of at-risk individuals” could be used as a platform for recruitment into trials testing preventive therapies and strategies. The overall aim of the RTCure project is to contribute to the development of therapies that affect the adaptive immune system via tolerization and other strategies, and to enable such therapies to be tested and implemented in very early phases of the disease, preferably even before joint inflammation has occurred. This endeavor is built on the premise that MSK complaints (i.e., stiffness, numbness, reduced function) are key symptoms that cause a person to seek medical advice, and therefore represent a feasible starting point for considering study enrolment. However, the terms “arthralgia” and “joint pain” can be used synonymously (we will use the term “arthralgia”) and inclusion in the register must therefore also be built on other criteria, which in the present setting is frequently the presence of autoantibodies to citrullinated proteins/peptides (ACPA) and/or rheumatoid factor (RF), but can also be a set of clinical and imaging determinants as described for the term “arthralgia suspicious for the progression to rheumatoid arthritis.” Other rheumatic and non-rheumatic causes should be ruled out before combining arthralgia with other factors to better characterize the at-risk state (i.e., morning stiffness, family history of RA, involvement of the metacarpophalangeal joints) (6). The risk of developing RA in such settings is being studied prospectively by several different European national and local initiatives.

In the current report, we outline consensus work to derive core outcomes of interest for a European registry of individuals at risk of RA, differences and parallels between those European initiatives it encompasses, and the challenges of building and managing such a registry.

## Methods

### Developing a Core Data Set to Collect in At-Risk Individuals

The RTCure Research Consortium, created in 2017 with funding from the IMI (“Innovative Medicines Initiative 2 Joint Undertaking”) assembled 20 academic and industry stakeholders as well as at risk individuals and patients with RA (www.RTCure.com) with the aim of earlier detection and prevention of RA. To help synchronize future work one major task was to define the populations with a risk to develop RA and to agree on a minimum core set of variables that should be collected and reported in a future mutual registry. During working group meetings from 2018 to 2020, different definitions were discussed and reassessed regarding feasibility with the RTCure partners interested in contributing to this common registry and with the aim to also include other partners once the registry has been established. The final set of variables was agreed upon together with the small-medium enterprise (SME) Zitelab, experienced in creating registries. Zitelab was then tasked with managing the infrastructure of the database.

### Set-Up of a Multi-Center Registry

One major emphasis in RTCure was to establish an infrastructure that will permit coordinated clinical trials of immunotherapy in seropositive individuals at-risk for RA, but yet without signs of joint inflammation. In a collaboration between several academic partners and the SME ZiteLab, an electronic registry has been developed that will allow consolidation of registries of individuals at-risk of developing RA across multiple academic partners. The registry platform is also a tool to help harmonize and balance the different reporting of collected variables. The contribution of ZiteLab is to provide an IT-based research infrastructure as an integral research partner of the collaborations (14, 15). All participating centers had received local ethical approval for their registry projects and additional documents by their respective legal departments for data sharing have been developed.

### Existing Cohorts and Recently Established Programs

We describe and compare cohorts of five RTCure partners (Karolinska Institutet, Medical University of Vienna, University Clinic Erlangen, Leiden University Medical Center and University of Newcastle). Established at different points in time, they represent overlapping groups amongst the heterogenous population of interest to RT-Cure. Communalities and differences are reported descriptively.

## Results

### Derived Core Outcomes for People At-Risk

Since 2018 members of the RTCure consortium have met on several occasions, together with the ZiteLab managers, and defined what types of data are recommended to be included in the registry. This was an iterative process, identifying commonalities between the existing site-specific registries, and further defining what data should be included in future prospective studies.

During discussion rounds at working group meetings, at conferences and via email, the core set of variables of highest interest was developed and finally agreed during the working group meeting at EULAR 2019 (Table 1). The pre-work of the EULAR TF on defining CSA was considered in the discussions concerning the construction of this set (6, 16). Including information on autoantibodies and eventual fulfillment, or the degree of fulfillment, of CSA criteria is of importance, since they are among the most promising target populations for preventing RA (4, 17, 18).

**Table 1:** Agreed minimum core set to report in individuals at-risk studies.

**Baseline/demographic characteristic parameters**			
Sex		Class of pain medication
Year of birth		Available biologic specimen
First rheumatic consultation		Height
Inclusion year		Weight
Symptom onset		Smoking
Type of patient consent		Alcohol
**Follow-up variables**			
Any treatment			
Laboratory Markers			
	C-reactive protein	
	Erythrocyte sedimentation rate		
	Anti-CCP	
	IgM rheumatoid factor	
Objective markers of disease activity			
	Swollen joint count 66	
	Tender joint count 68	
	Disease activity score	
	CDAI	
Patient-reported outcomes			
	Pain	^*^
	Fatigue	
	Patient global assessment		
	Health assessment questionnaire		
	Morning stiffness	
	Day/time of most severe symptoms		
Evaluator global assessment			

### One Database for Different At-Risk Cohorts

The efforts toward data harmonization have led to the construction of a functional registry and web-based interface. The technical backbone of the RTCure at-risk-registry was built between 2018 and 2019. During a 2-day consensus event, with participation from each of the involved centers, an extended list of all candidate-variables was described with a reference to its use in either EULAR recommendations, prior decisions of the consortium or existing (or planned) data collections in the participating centers. In a consensus-process, each variable was given an importance-score between level 1 and 4 (4 being the lowest). Variables that were assigned an importance-score of one eventually were then termed as the core set variables. During the consensus event, an information technology (IT) system was built resulting in a web-based system where each of the centers—having secure access to their own part of the system—can see the decisions expressed within metadata sheets of variables, upload facilities with validation of uploads, web entry forms and search facilities (19). In the development phase all variables suggested were included in a first version. Then based on further dialogue every variable was tagged with an importance score.

Growing with the needs of the collaborators, the structure was continuously fine-tuned with improved access to a metadata-sheets explaining details of the data-model, hence the variables, types and parameter values. Furthermore tools for dialogue-oriented clarifications of potential differences between the agreed data-model built in the system and the available local data of each partner has been put in place. Since the database tool and the dialogue and the metadata sheet validating the upload are based on the same excel sheet (automatically parsed into different use-cases) the process was highly efficient. By using the approach of an “importance scores” still the database contains a part where all participating partners agree and other parts where variables are not used or shared by all partners. In early 2022 a tool for accessing summary data across the different cohorts is being added.

This early and instant availability of a functional IT-system was based on reuse of components from a pre-existing IT-system used by the global myositis community (14) and EuroSpA collaboration (15).

During the following months the main barrier to overcome related to establishing GDPR-compliant risk assessments which conformed with the different traditions between the various participating University Hospitals, and Data Processor Agreements (DPA) between the University Hospitals and ZiteLab as a pre-condition for data uploads.

An important challenge, even within a consortium, was overcoming ethical and legal requirements to be able to upload data into the registry. Ethical issues are different in every country due to country-specific procedures and because of different underlying data-collections from which the data-extractions are done. Legal issues relate to (i) different procedures in risk-analysis, (ii) different classification related if data processing agreements are needed, (iii) capacity problems in the legal units and (iv) unclarified internal data transfers agreements between university units and hospital units. The legal implementation strategy involved first establishing a completed risk assessment and DPA-agreement with one of the University Hospitals and then rolling out the same set of documents to the remaining centers, with slight adaption to local regulations and governance. As example in Sweden clarification of the related role of University Hospital and the Research Institute, In Germany the relation to Secrecy Obligation according to § 203 German Criminal Code (StGB). In the United Kingdom the Data Protection Impact Assessment had to use an extended template.

During a period of 2 years these negotiations led to contracts with the cohort partners and uploads of datasets from two partners, with expected completion of the full process of first data uploads in the first quarter of 2022.

### Patient and Public Involvement

Patient Research Partners (PRPs) are defined as “persons with a relevant disease who operate as active research team members on an equal basis with professional researchers, adding the benefit of their experiential knowledge to any phase of the project” (20). Two PRPs with established RA from the UK were involved with the set-up of this registry and attended registry-specific discussions during the EULAR annual congresses and the RTCure annual meetings from 2018 onwards. At the EULAR 2018 meeting, one PRP questioned why seronegative people were not going to be included strategically in the registry database. Following a discussion between all stakeholders, it was decided that although the emphasis as prospective entry criterion is set on seropositivity, all data in existing registries would be included to the RTCure at-risk-registry independent of detected autoantibodies.

### European Cohorts: Mutual Aims and Differences

#### Karolinska Institutet

At the Karolinska Institutet and the Karolinska University Hospital, the Risk RA prospective research program to study individuals with MSK symptoms and systemic autoimmunity, specifically ACPA has been established in year 2015. It aims to understand how symptoms and biomarkers evolve over time in individuals who develop arthritis within 3 years compared with those who do not. Particular emphasis lies on the development of predictors for ACPA-positive arthritis development and on the presence of pain and fatigue over time in individuals in the cohort. The program includes assessing the impact of genetics and environmental and lifestyle factors on the risk for arthritis development and on the symptomatology during the observation time, using an extensive questionnaire for lifestyle and environment and genome-wide association studies (GWAS)-based genetic analysis. The RISK RA cohort encompasses about 300 people, who are followed up through the RISK RA register over 3 years in a predefined schedule or until the onset of arthritis (Table 3). The individuals are identified in primary care as individuals with MSK complaints, suspicious for a rheumatic disease and referred to the rheumatology clinic. At the clinic, clinical and ultrasound (US) examinations are performed to evaluate joint inflammation. Tendon and bone involvement is additionally checked by means of US but not taken into account for deciding whether an individual would be included or not. If any signs of joint inflammation (arthritis on either clinical or US investigation) are identified, the person is diagnosed with arthritis in need of immediate treatment and is followed-up according to existing national and international guidelines. To be able to study the risk of arthritis onset, strict inclusion criteria (Table 2) are applied for the program making only individuals with minimal US changes able to participate. Hence, individuals scoring > 1 by gray scale and/or ≥ 1 by power Doppler using the EULAR-OMERACT scoring system (21) are excluded from participation. Individuals that do not have signs of joint inflammation are then invited to participate in the RISK RA program. Neither before inclusion or during the follow-up individuals are allowed to receive glucocorticoids (GC) or DMARDs. During the program individuals are advised on symptomatic pharmacological (NSAIDs) and non-pharmacological treatments (e.g., physiotherapy). The follow-up strategy includes both on demand rapid visits if symptoms worsen and routine follow-ups with at least yearly visits. If clinical or US arthritis develop during the follow-up period, people are treated according to the national guidelines for RA treatment.

**Table 2:** Criteria necessary for inclusion into the individual at-risk cohort programs.

**Criteria**	**KI**	**MUV**	**UKER**	**LUMC**	**UNEW**
CCP positivity	+	±	+	±	+
RF positivity	±	±	+	±	±
CCP AND/OR RF positivity	±	+	±	±	±
EULAR CSA criteria fulfilled	±	±	+	±	±
Clinical suspicion by a rheumatologist^*^	+	+	+	+	±
No glucocorticoids reveived	+	+	+	+	+
SJC 66 = 0	+	+	+	+	+
No clinical arthritis	+	+	+	+	+
No synovitis detected by using US	+	±	±	na	±
Presence of tenosynovitis in US	±	±	±	na	±
Structural changes in US	±	±	±	na	±

*+, criteria must be fulfilled, ± is allowed to be fulfilled; na, not assessed*.

*CSA, Clinical suspect arthralgia for progression to RA;*

* *Meaning that an experienced rheumatologist concludes based on the assessment that this patient is at risk to progress toward the development of rheumatoid arthritis; KI, Karolinska Institute; MUV, Medical University of Vienna, in regard to the ASPRA cohort; UKER, University Clinic Erlangen; LUMC, Leiden University Medical Centre; UNEW, Newcastle University*.

#### Medical University of Vienna

##### PRERA

The PRERA cohort is a closed cohort without further recruitment or follow-up. This study was conducted between 2010 and 2018. Included individuals were followed-up over 5 years. None of them developed classifiable inflammatory arthritis. People were enrolled *via* the Austrian free annual health examination, independent of the presence or absence of MSK symptoms, but excluding those with established inflammatory rheumatic conditions. Seropositive individuals were matched for sex and age with seronegative individuals and have undergone assessment of RF, ACPA, RA-33, lifestyle and family history at baseline and routine clinical and laboratory assessments every 6 months. Table 3 reports on data of 98 individuals that accepted the invitation to participate. Of note, the rates of progressors reported in Table 3 relate to swollen joints due to any reason and should not be interpreted as progression toward RA. However, since only 45 individuals continued within this program after 2 years, it is to be expected that in the healthy asymptomatic drop-out population, no rheumatic condition so far manifested.

**Table 3:** Overview of risk cohorts included in the RTCure at-risk registry infrastructure, with follow-up data in July 2021.

		**KI**	**MUV—PRERA**	**MUV—ASPRA**	**UKER**	**LUMC**	**LUMC (ACPA+)**	**UNEW**
Number		268	98	28	106	645	91	32
Age	median (iqr)	48 (36–58)	57 (47–64)	53 (40–57)	50 (40–58)	44 (34–54)	52 (39–57)	50.5 (34–57)
Female sex	*n* (%)	212 (79)	55 (61)	22 (78)	73 (68.9)	490 (76)	72 (79)	22 (69%)
Symptom duration (months)	median (iqr)	22 (10–50)	missing	14 (6–12)	37.5 (26.8–96)	4 (2–9)	5 (3-12)	4 (2–9)
Ever smoked	*n* (%)	150 (58)	51 (56.7)	10 (59)	59 (55.7)	326 (58)	56 (71)	16 (50%)
Never smoked	*n* (%)	110 (42)	36 (36.7)	7 (41)	42 (39.6)	237 (42)	23 (29)	3 (9%)
Current smoker	*n* (%)	44 (17)	24 (24.0)	6 (35)	35 (33.0)	120 (21)	24 (30)	9 28%)
Previous smoker	*n* (%)	106 (41)	29 (30.0)	4 (24)	24 (22.6)	206 (37)	32 (41)	7 (22%)
Pain (VAS, 0–100)	median (iqr)	26 (10–52)	0 (0-9.5)	5 (2.5–7)	17 (3–33)	5 (3–7)	4 (2-6)	40 (0–70)
Patient Global Assessment (VAS, 0–100)	median (iqr)	28 (6–51)	0 (0–5)	3 (2–7)	12 (1–33)	3 (2–5)	3 (1–6)	37 (20–70)
Evaluator Global Assessment (VAS, 0–100)	median (iqr)	0 (0–1)	0 (0–0)	1 (0–2)	3 (2-12)	missing	missing	10.5 (1-57.5)
Morning stiffness ≥ 60 min	*n* (%)	57(28)	0 (0%)	3 (13)	9 (8.5)	212 (35)	30 (35)	9 (28)
SJC28	median (iqr)	0 (0–0)	0 (0–0)	0 (0–0)	0 (0–0)	0 (0–0)	0 (0–0)	0 (0–0)
TJC28	median (iqr)	0 (0–2)	0 (0–0)	2 (0–5)	0 (0–2)	3 (1–6)	2 (0–3)	1 (0–3)
CRP (mg/dl)	median (iqr)	0.1 (0.1–0.4)	0.15 (0.07–0.27)	0.145 (0.12–0.26)	0.52 (0.39–0.55)	0.30 (0.30–0.47)	0.36 (0.30–0.72)	0.4 (0.4–0.6)
ESR (mm/h)	median (iqr)	11 (5–19)	10 (6–15)	11 (7–20)	11.5 (7–17)	6 (2–14)	11 (6–24)	8.5 (5–15.25)
Frequency anti-CCP positivity	*n* (%)	268 (100)	3 (3.3)	6 (37.5)	84 (79)	91 (14)	91 (100)	32 (100)
Anti-CCP titre (times relative cut-off)	median (iqr)	10 (3–100)	0.8 (0.4–1.9)	1.8 (0.7–136)	88.8 (15.8–1574.4)	0.14 (0.10–0.14)	29 (7–49)	233.5 (20.5–301)
Frequency RF positivity	*n* (%)	33% (88)	27 (30)	14 (87.5)	60 (56.6)	135 (21)	70 (77)	21 (65)
RF titre (times relative cut-off)	median (iqr)	0 (0–1.6)	0 (0–11.6)	35 (18–48)	22 (11.6–62.8)	0.23 (0.11–0.66)	5 (1–18)	61 (38–130.5)
Follow-up time (months)	median (iqr)	19 (12–26)	24.5 (6.8–55.0)	4 (2–6)	2 (1–5)	24 (11–26)	4 (1–23)	44 (28–82)
Arthritis progressors 0–6 months	*n* (%)	26 (10)	7 (9)	4 (14)	22 (21)	73 (13)	36 (50)	7 (22)
Arthritis progressors 0–12 months	*n* (%)	41 (17)	8 (12)		27 (26)	77 (14)	37 (51)	9 (35)
Arthritis progressors 0–24 months	*n* (%)	67 (44)	10 (22)		34 (36)	88 (17)	41 (59)	13 (52)
Ever arthritis progressors	*n* (%)	75 (28)	10 (10)	4 (14)	41 (38)	98 (15)	44 (48)	17 (53)

*KI, Karolinska Institutet; MUV, Medical University of Vienna; UKER, University Clinic Erlangen; LUMC, Leiden University Medical Centre; UNEW, Newcastle University; VAS, Visual Analogue Scale; TJC, Tender Joint Count; SJC, Swollen Joint Count. **(**Progression rates of PRERA relate to swollen joints due to any reason and not to progression to RA)*.

##### ASPRA

The Vienna Arthralgia Suspicious for Progression to Rheumatoid Arthritis (ASPRA) registry was started in August 2020 within a specialized outpatient program of the MUV, including seropositive individuals with arthralgia, without clinical arthritis in a structured follow-up management program. The ASPRA program is already based on the agreed core data for the meta-database originated in the RTCure project and data on radiologic changes (assessed by US and micro computed tomography—micro-CT), as well as lung function and cardiovascular risk factors which are longitudinally collected in addition. Inclusion in the program is based on the presence of positive CCP or RF tests in individuals with MSK complaints, without clinical arthritis, but the suspicion of the rheumatologist for the risk of progressing to RA (Table 2). At-risk individuals are invited to remain in the program over 5 years with visits twice yearly or until the onset of any classifiable rheumatic condition. All study participants complete questions on CSA (6), the SPARRA questionnaire (22) and take part in the biobanking program of the division for retrospective analyses of molecular targets of interest. GCs or DMARDs cannot be received before inclusion or during the at-risk phase. Similarly to the program at KI symptomatic therapy is offered. In comparison to the historical PRERA study that invited seropositive and control individuals without the need of symptoms recruited through referrals from yearly health check-up offered by the public health system, this ASPRA registry has a higher potential to identify individuals who go on to develop arthritis. Around 1 year after start of this program 4 out of 28 patients have developed classifiable RA (Table 3).

#### University Clinic Erlangen

To explore the development of arthritis, a RA at-risk cohort (IRACE cohort Individuals at Risk for Arthritis Cohort Erlangen) was initiated in 2011. This prospective cohort includes people with serological evidence of CCP antibodies with or without MSK symptoms (Table 2). Two participants are without MSK symptoms but only have rheumatoid nodules. Individuals with clinically apparent arthritis (at least one swollen joint with synovitis in clinical assessment in the 66 joint count, performed by an experienced rheumatologist) are excluded. Currently, the RA-at-risk cohort consists of 175 at-risk individuals (as of November 2021). Table 3 provides an overview on participant characteristics with follow-up time longer than a year. Individuals with permanent GC therapy or GC therapy at the time of initial presentation are not included into the cohort. Individuals within the program should not require GC at the time point of a visit. However, intake of GCs in between visits for no more than 2 days is permitted. Participants are seen between every 3, 6, or 12 months during their clinical routine appointment. After informed consent, basic characteristics such as body mass index, periodontitis, family history of RA, medication, comorbidities, alcohol intake, smoking as well as 66/68 swollen/tender joint count are obtained. Visual analogue scales (VAS; scores of participants and physicians), DAS28 and Health Assessment Questionnaire (HAQ) are available. For basic research purposes, serum and full blood samples are collected and stored. Endpoints are: ACR-EULAR Classification criteria/clinical apparent arthritis. Timespan between the first symptom that led to a person seeking healthcare to diagnosis is calculated. Within the at-risk program, individuals should further be recruited to interventional trials. All receive symptomatic (NSAIDs) therapy as required, as well as non-pharmacological treatment intervention, like education on life-style and diet.

The strength of this prospective observational cohort is the systematic linkage of clinical data with serological biomarkers and state-of-the-art MSK imaging, such as MSK US, HR-pQCT (high resolution peripheral quantitative computed tomography) 1.5 Tesla, high-field 7 Tesla magnetic resonance (MRI) or innovative metabolic imaging approaches such as Fibroblast activation positron emission tomography (FAPI-PET) as in current research projects (13, 23–30). This cohort serves as a source for prospective interventional trials such as the ARIAA trial (EUDRA-CT 2014-000555-93) to study abatacept in the context of preventing or delaying disease onset in RA-at risk individuals with subclinical signs of inflammation as judged by MRI (31). The primary endpoint of this trial, which involves a 6-months treatment phase with abatacept or placebo as well as 12 months follow-up, is defined as an improvement in at least one of the assessed MRI inflammation parameters. Additional questions that are addressed include the progression to clinically overt arthritis of these at-risk individuals upon abatacept treatment and during the follow up period.

#### Leiden University Medical Centre

The Leiden Clinically Suspect Arthralgia cohort includes consecutive patients presenting with CSA. People with arthralgia of the small joints for <1 year that is considered suspicious for progression to RA are included at their first visit to the outpatient clinic, thus before any blood tests have been performed (Table 3). People are not included if the rheumatologist considered another explanation for their arthralgia (e.g., osteoarthritis or fibromyalgia) more likely than imminent RA. Presence of clinical arthritis (joint swelling) also precludes CSA by definition. In line with national guidelines for general practitioners (GPs), GPs are discouraged to perform ACPA-testing themselves but are encouraged to refer patients in case of any suspicion of imminent RA. Hence, inclusion is mostly done without knowledge of the results of additional investigations (Table 2). Treatment with GCs is not allowed before entering the program or during follow-up. Follow-up visits are performed at 4, 12, and 24 months and more regularly in case of increased symptoms. During follow-up, CSA-patients are not treated with DMARDs or GCs but symptomatic treatment with NSAIDs or analgesics is possible. At each study visit patient-reported outcome questionnaires are completed, physical joint examination performed and blood samples taken. In addition, an MRI of small joints is conducted at baseline. Patients are followed for development of clinical arthritis, confirmed with joint swelling at physical examination by the rheumatologist. Fulfillment of classification criteria is noted. This strategy allows to include both autoantibody positive and autoantibody negative RA in the pre-arthritis stage of RA (32).

#### Newcastle University

The Newcastle “At-Risk of RA” cohort is a defined sub-cohort within the Northeast Early Arthritis Cohort (NEAC)—itself an unselected, observational inception cohort of consecutive, consenting individuals referred from primary care to the Suspected Inflammatory Arthritis service at Newcastle Hospitals NHS Foundation Trust (Table 3). Primary care physicians are encouraged to refer such individuals without the need to undertake blood tests (including autoantibodies), since these are routinely performed during secondary care assessment. All such individuals receive two initial assessment appointments 1 week apart. At the first visit, recording of detailed baseline demographic and clinical parameters is undertaken, along with MSK US assessment. At the subsequent visit, people are reviewed by a consultant rheumatologist with access to all results, and assigned an initial clinical diagnosis from a dropdown menu of possibilities that includes “ACPA+ arthralgia.” Individuals placed in this category by their consulting rheumatologist (confirmed to have 0 recorded swollen joints out of a total of 74 assessed and a positive anti-CCP2 test result according to routine laboratory testing are defined eligible for inclusion in the “At-Risk of RA” NEAC sub-cohort (Table 2). Enrolled individuals are subject to routine care with clinic visit frequency and treatment is at the discretion of their consulting rheumatologist. Treatment with GCs, as well as DMARDs prior to enrolment are not allowed and during follow-up until development of clinical arthritis GCs are discouraged and DMARDs not prescribed. Follow-up data including tender/swollen joint counts and VAS are recorded, data being routinely captured through the electronic patient record (EPR) which is linked to a bespoke database at Newcastle University for research purposes. There is an opportunity for biological sampling for research at baseline and follow-up (33). The development of arthritis represents the end-point (defined as joint swelling confirmed by a rheumatologist); fulfillment/non-fulfillment of RA classification criteria is also recorded for purposes of the current registry, and any immunomodulatory therapeutic interventions are also logged. The overall strategy ensures that prospective data reflect routine, consultant-led care at a single center (34).

### Synthesis of Cohorts

**Figure 2:**
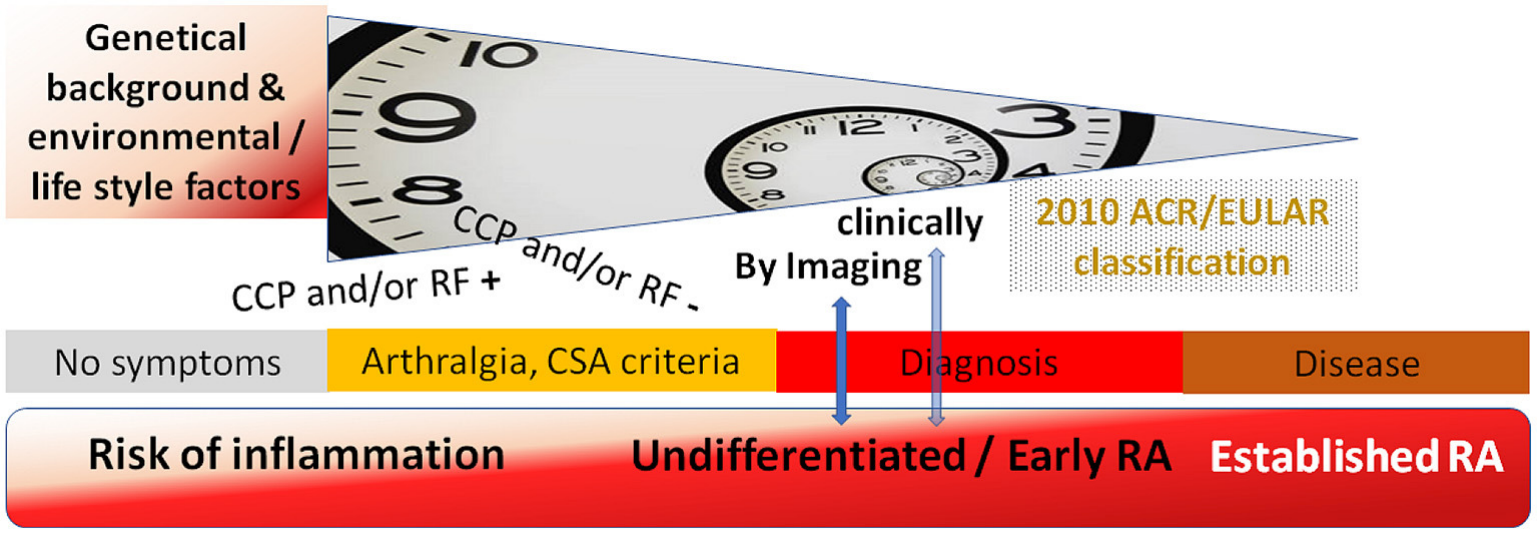
Overview of study time points in the at-risk programs, following up antibody (CCP and/or RF) positive and negative individuals until occurrence of arthritis detected by imaging (KI) or clinically (MUV, UKER, LUMC, UNEW), potentially with overlap to classification as RA according to the 2010 ACR/EULAR criteria.

These cohorts represent different stages of the longitudinal development of RA with broad clinical characterization and sample collection, allowing in-depth studies on the immune events responsible for each of these stages (Figure 2). Data on environmental exposures (specifically smoking) and genetic predisposition (genotyping) as well as biological samples (including blood cells, serum, DNA and RNA preparations) are available for most of the individuals in the cohorts. In selected individuals, we also have access to tissue samples including mucosal samples. The described data is already available in some of the cohorts (such as antibody testing and genotyping in the Karolinska RISK RA cohort and the Leiden early arthritis clinic). This heterogeneity at inclusion as well as the individual cohort endpoints can be overcome by harmonized data reporting. For example, the individuals with clinical arthritis in the at-risk cohort at KI can be pooled with those of other cohorts, since all cohorts contain data on the occurrence of clinical arthritis. When we apply the same timeframe of follow-up results, we can see that the arthritis progression rates across the cohorts become increasingly similar with the seropositive individuals showing progression rates between 44% and 59% within 2 years of follow-up. Following the dogma of harmonized reporting, individuals across all cohorts can be divided into subgroups either fulfilling the criteria of CSA, or overlap of presence for predefined autoantibodies or imaging features at time of inclusion. By collecting this within the RTCure at-risk registry, information regarding samples sizes of different sub-groups can be checked easily, quickly and conveniently.

## Discussion

Today, no therapies are approved specifically for the treatment of clinical syndromes that precede RA in individuals with early evidence of autoimmunity, or for the prevention of RA. An important objective in studying at-risk individuals is to enable accurate identification of those with certain types of immune reactions and other biomarkers associated with clinical symptoms that together suggest a high-risk state for progression to clinically evident RA. One major aim of the described registry was to create an infrastructure, *via* a European collaborative network, that can catalog cohorts of such symptomatic patients at high risk of RA who could be enrolled into clinical trials aimed at preventing progression to RA. A specific goal is to create opportunities to selectively target therapies against disease-inducing immune reactions. The program also enables a more rapid diagnosis of arthritis in those individuals included in the register who have regular contact with rheumatologists. Here, it will be possible to eliminate most currently encountered patient-related and doctor-related delays in the diagnosis and treatment of arthritis, enabling intervention for undifferentiated inflammatory arthritis very early, with the aim to prevent progression to the disease classified as RA.

In order to further develop this concept from a research-based registry to routine care, it is paramount to work together between several different stakeholders with patients with RA and individuals at-risk for developing RA. This requires close collaboration between academia, care providers and industry: all these networks exist within the RTCure consortium and will feed into the registry's outputs including clinical trials and, ultimately, implementation of guidelines for prevention of RA in widespread clinical practice.

A fundamental laboratory finding that provides a scientific basis for the RTCure program and the registry, is that development of RA-specific autoantibodies against proteins/peptides post-translationally modified by citrullination (ACPA) or other modifications (collectively named AMPAs) precede the onset of joint inflammation by many years. An increase in titers, epitope spreading and autoantibody isotype switching occurs before onset of joint inflammation; conversely, very few patients “seroconvert,” i.e., develop autoantibodies, after onset of disease (35–37). The presence of ACPAs also coincides in most cases with the presence of rheumatoid factors (RF) at diagnosis and the disease subset positive for either ACPA or RF, or both, is nowadays conventionally labeled as “seropositive RA.” Notably, in the risk phase there seem to be a higher proportion of ACPA positive individuals that are RF negative. Once at diagnosis relatively few patients seroconvert from positive to negative during treatment (38). These and other observations have underpinned concepts of autoimmunity in the aetiopathogenesis of RA, implying causality. Autoantibodies like ACPA are components of immune complexes capable of activating immune effector cells (e.g., osteoclasts) to trigger pathological reactions (bone loss) which in turn leads to clinical symptoms (13, 39, 40). Furthermore, seronegative patients are in many cases not truly seronegative. ACPA fine-specificities, IgG/IgA RF or anti-carbamylated, as well as other AMPAs can be found in some individuals negative for routine anti-CCP and/or RF tests. It seems that the HLA-DRB1 SE is associated with the formation of ACPAs, whereas smoking has its major role in individuals positive for both RF and ACPA, some indications highlight smoking with the occurrence of RF in seronegative disease (41, 42).

All the described programs that are summarized within the RTCure at-risk registry have set different emphasis on different research questions. This can be seen as advantageous for exploring the pathway of at-risk individuals holistically. One crucial point in these programs is the embedment into structures of the healthcare systems of the respective countries/regions to represent the local standard of clinical care that might help in having at-risk individuals feel more comfortable in this limbus of maybe developing or not developing the disease (5). Every cohort-specific approach for setting inclusion and exclusion criteria has advantages, when taking local circumstances into account. The selection of only at-risk individuals with CCP positivity homogenize individuals under observation (genetic and environmental exposure), leading to higher observed progression rates, but is not representative for all patients with RA that are currently treated in usual care (43). Setting less stringent inclusion criteria (e.g., not mandating seropositivity and/or the detection of subclinical inflammation) allows answering questions of common rheumatoid arthritis symptoms (both seropositive and seronegative variant) and assessments (44). However, from a helicopter perspective we can better grasp that our current problem is to investigate many factors taken together for derivation of better prediction estimates. This poses a challenge for individual cohorts and can only be addressed by studying combined data in a concerted effort.

The cohorts and the European RTCure at-risk RA Registry will allow identification of candidates for clinical trials by screening within the core data set assessments that the partners have agreed on. Such studies are already ongoing (the Treat Earlier study using methotrexate in people with CSA with subclinical joint inflammation in the Netherlands, the APRIPPA study using abatacept in people with seropositive arthralgia in the UK, the ARIAA study using abatacept to treat seropositive arthralgia in Germany and the PREVENT RA study using bisphosphonates to treat pain in seropositive individuals with MSK complaints in Sweden). Currently two thirds of patients fulfilling RA disease classification criteria are characterized by the presence of autoantibodies that have been post-translationally modified. As more disease-relevant post-translational modifications are discovered, the proportion of seropositive RA patients will likely increase (42). This means that in the future more autoantibody-positive at-risk individuals could be eligible for intervention studies. Detecting these autoantibodies early in the disease course has clinical value in at-risk cohorts since they identify individuals at highest risk of developing RA (45).

Only recently, the European Alliance of Rheumatology (EULAR) has published points to consider for conducting clinical trials and observational studies in at-risk individuals (46). The different outcomes for assessment agreed on within our consortium are also named in this recommendation paper, which also reflects the different phases in the development of arthritis, and the foci that are set in the contributing individual registries. As in every collaborative registry also the RTCure at-risk RA Registry is not fully populated with all entries of variables available but has agreed on a core set to work with considering also availability and feasibility. Relevant Items, like ethnicity and family history may also be reported in this registry, but at this point have not been deemed sufficiently available and of interest to the overlap of all processed items among the individual cohorts. Our registry remains dynamic and welcomes further collaboration and inclusion, which over time logically leads to adaptations of the core set.

Taken together, the register for at-risk for RA individuals and the harmonization of clinical data, biobanking and “omics” data for risk estimation, patient stratification and disease monitoring, will provide an internationally unique resource for understanding the longitudinal development of RA (Box 1), and also provide the pharmaceutical industry and academia with a potential to conduct clinical trials intended to prevent the development of RA.

Box 1 **Research agenda.**

- Better understanding of the trajectory to RA, in dependency of different phases or substages
  ° Understanding of the role of risk factors in symptomatic individuals without signs of inflammation in imaging and with signs of inflammation in imaging
  ° Differences in risk factors between ACPA positive and negative individuals, and evaluation of a common path
- Developing of a validated risk stratification method to be used in clinical practice to support future trials and to support communication with regulatory agencies
- Defining the different outcomes and their interrelationships: imaging arthritis, clinical arthritis, clinical arthritis that remits spontaneously, persistent clinical arthritis
- Defining relevance of changes in regularly assessed outcomes in at-risk programs
- Contributing to clinical trials aimed at prevention of arthritis and alleviation of symptoms in the at-risk phase
- Defining at-risk Individuals view of acceptable risk/risk period to consider preventative treatment in the development pathway of RA

## Data Availability Statement

The original contributions presented in the study are included in the article/supplementary materials, further inquiries can be directed to the corresponding author/s.

## Ethics Statement

The studies involving human participants were reviewed and approved by Ethical Committee of the Medical University of Vienna, Ethical Committee of Stockholm, Ethik-Kommission der Friedrich-Alexander-Universität Erlangen-Nürnberg, Medical Ethical Committee of the LUMC and Newcastle and North Tyneside 2 Research Ethics Committee.

## Author Contributions

PS, AH, AK, AH-vM, AP, DS, GK, MJ, NK, LK, and AC were involved in data collection, handling, and interpretation. The manuscript was drafted by PS and complemented by input from all authors. All authors contributed to the set-up of this project. The final version was approved by all authors.

## Funding

The RTCure project has received funding from the Innovative Medicines Initiative 2 Joint Undertaking under Grant Agreement no. 777357. This joint undertaking receives support from the European Union's Horizon 2020 Research and Innovation Programme and EFPIA. PS has been supported by the FOREUM research fellowship grant. The Newcastle (NEAC) cohort receives infrastructural support *via* the National Institute for Health Research Newcastle Biomedical Research Centre.

## Author Disclaimer

The views expressed are those of the author(s) and not necessarily those of the NHS, the NIHR or the UK Department of Health.

## Conflict of Interest

The authors declare that the research was conducted in the absence of any commercial or financial relationships that could be construed as a potential conflict of interest.

## Publisher's Note

All claims expressed in this article are solely those of the authors and do not necessarily represent those of their affiliated organizations, or those of the publisher, the editors and the reviewers. Any product that may be evaluated in this article, or claim that may be made by its manufacturer, is not guaranteed or endorsed by the publisher.
